# RBBP4 Enhances Platinum Chemo Resistance in Lung Adenocarcinoma

**DOI:** 10.1155/2021/6905985

**Published:** 2021-01-09

**Authors:** Nianwu Wang, Wei Wang, Wenli Mao, Nazuke Kuerbantayi, Nuan Jia, Yan Chen, Fang Zhou, Li Yin, Yukun Wang

**Affiliations:** ^1^School of Medicine, Southern University of Science and Technology, Shenzhen, Guangdong 518055, China; ^2^Department of Pharmaceutics and Pharmacy Administration, School of Pharmacy, Air Force Medical University, Xi'an, Shanxi 710032, China; ^3^Southern University of Science and Technology Hospital, Shenzhen, Guangdong 518055, China; ^4^Key Laboratory of Tropical Translational Medicine of Ministry of Education and School of Tropical Medicine and Laboratory Medicine, Hainan Medical University, Haikou, Hainan 571199, China

## Abstract

**Background:**

The majority of lung cancers are adenocarcinomas, with the proportion being 40%. The patients are mostly diagnosed in the middle and late stages with metastasis and easy recurrence, which poses great challenge to the treatment and prognosis. Platinum-based chemotherapy is a primary treatment for adenocarcinoma, which frequently causes drug resistance. As a result, it is important to uncover the mechanisms of the chemoresponse of adenocarcinoma to platinum-based chemotherapy.

**Methods:**

The genes from the dataset GSE7880 were gathered into gene modules with the assistance of weighted gene coexpression network analysis (WGCNA), the gene trait significance absolute value (|GS|), and gene module memberships (MM). The genes from hub gene modules were calculated with a protein-protein interaction (PPI) network analysis in order to obtain a screening map of hub genes. The hub genes with both a high |GS| and MM and a high degree were selected. Furthermore, genes in the hub gene modules also went through a Gene Ontology (GO) functional enrichment analysis.

**Results:**

11 hub genes in four hub gene modules (LY86, ACTR2, CDK2, CKAP4, KPNB1, RBBP4, SMAD4, MYL6, RPS27, TSPAN2, and VAMP2) were chosen as the significant hub genes. Through the GO function enrichment analysis, it was indicated that four modules were abundant in immune system functions (floralwhite), amino acid biosynthetic process (lightpink4), cell chemotaxis (navajowhite2), and targeting protein (paleturquoise). Four hub genes with the highest |GS| were verified by prognostic analysis.

## 1. Introduction

Lung cancer has become the malignant tumor with the highest morbidity and mortality in the world. It is estimated that 1.8 million people are diagnosed with this disease and 1.6 million die each year, which has shown a sharp increase [1]. Due to the heterogeneity of lung cancer tissues, the complexity of cellular, molecular, and genetic characteristics and immune status, the treatment effect is not ideal with a 5-year survival rate of less than 20% [1]. Lung cancer can be divided into small-cell lung carcinoma and non-small-cell lung carcinoma (NSCLC) according to the clinical treatment combined with biological characteristics. The NSCLC is one of the most noticeable types of lung cancer, which contributes to almost 85% of them [2]. The most common types of NSCLC are lung squamous cell carcinoma (LUSC) and lung adenocarcinoma (LUAD), of which adenocarcinoma is considered the most common type of lung cancer with the proportion in all lung cancer cases being 40% [3]. Most patients with LUAD are diagnosed in the advanced stage, often accompanied with metastasis, resulting in increased treatment difficulty and a poor prognosis. Currently, the main clinical treatment methods for lung cancer involve surgery, chemotherapy, radiotherapy, antiangiogenesis inhibitors, and targeted drugs. Chemotherapy is a common treatment for NSCLC, given that chemotherapeutic drugs gemcitabine, pemetrexed, and paclitaxel can be used along with platinum-based drugs such as cisplatin, carboplatin, and nedaplatin [4]. However, it could result in drug resistance and the recurrence of tumors [5]. In order to better understand and solve the side-effects, the mechanism of gene regulation related to the chemoresponse of adenocarcinoma to platinum-based chemotherapy still needs to be explored.

Based on bioinformatics to study the gene expression microarray, this study is aimed at exploring hub genes associated with recurrent LUAD despite platinum-based therapy. A matrix of gene expression dataset (GSE7880) with recurrent adenocarcinoma samples was utilized for the hub genes. After analyzing the correlative level of each gene expression, the genes with a high level of coexpression were analyzed and gene coexpression modules were established by weighted gene coexpression network analysis (WGCNA) [6]. With the help of a protein-protein interaction network (PPI network) [7] analysis, the hub genes could be screened. Furthermore, Gene Ontology (GO) functional enrichment analysis [8] was performed on genes in the gene modules to determine which module was directly associated with LUAD recurrence. Consecutively, the gene with the highest gene trait significance in the selected module was regarded as the final hub gene and verified by prognostic analysis.

## 2. Materials and Methods

### 2.1. Preparation of Dataset

A microarray dataset (GSE7880) retrieved from the GEO database was adopted in this study. The dataset GSE7880 was uploaded by Rohrbeck et.al, including mRNA expression profiles of 43 patients with NSCLC, among which 25 were diagnosed with adenocarcinoma and 18 with squamous cell carcinoma. The 25 adenocarcinoma patients were selected as the research objects, including 10 primary untreated adenocarcinoma and 15 recurrent adenocarcinomas in stage IIIB or stage IV. In addition, all the patients were treated with platinum-based therapy, while the recurrent patients were regarded as “non-response” with progression under platinum-based therapy. Tumor samples were isolated by laser capture microdissection, and Human HG Focus Array and Affymetrix were utilized for analysis expression data with 8793 defined genes in matrix. However, in the original matrix, there was no exact number for GSM190993, but other sample data was reserved with two decimal places. As a result, two decimal places for the data were adopted in GSM190993.

### 2.2. Construction of Weighted Gene Coexpression Network

WGCNA R package was utilized for the construction of a gene expression network which can find the gene modules where genes are synergistically expressed and explore the association between the network and the related phenotype [9]. This algorithm is based on the assumption that the gene network obeys scale-free distribution. Afterwards, gene expression matrix and trait matrix are inputted before the definition of the adjacency function in gene network form. Following that, the scale independence and mean connectivity are calculated and the power value is selected. Finally, the hierarchical clustering tree can be constructed. The data of adenocarcinoma patients in dataset GSE7880 were used as the gene expression matrix. In the trait matrix, the patients responded to the therapy were defined as “1” and the patients with “non-response” under platinum-based therapy was “0,” which could be used to construct the coexpression network to screen out the platinum chemoresistance-related hub genes. In regard to checking for any missing value, we started the filtration of the expression values using the mean, where “meanFPKM = 0.5” was set, and then two matrixes were inputted to do the sample clustering. To determine the soft threshold, a scatter plot of exponential and power value was made and the powers were set as 1 to 20 and the ab line of *R*^2^ was chosen as 0.8. For the gene clustering, the minimal number of the gene modules was set as 30. Then, the modules would be merged with the cut height equal to 0.25. Subsequently, a figure of merged clustering gene modules with dynamic tree could be gained, which contained 22 modules. In accordance with the match between the modules and chemoresponse, a module trait relationship diagram was obtained, which was used in the identification of hub gene modules. Finally, data which fulfilled Cytoscape were derived to make preparations for the PPI network analysis.

### 2.3. Identification of Platinum Based Therapy Response Related Module

The module eigengenes (MEs) were shown in the module trait relationship diagram which was considered the foundation for screening hub gene modules. With the assistance of Pearson's correlation test, the relative levels between MEs and platinum-based resistance were calculated. The MEs whose relative levels were higher than 0.4, and *p* values less than 0.05 were selected as the hub gene modules for further study.

### 2.4. Hub Gene Identification

During the gene coexpression network analysis, the gene trait significance absolute value (|GS|), which represented the relative level between genes and trait, and gene module membership (MM), which represented the contribution of the gene to this module, were calculated by the absolute value of Pearson's correlation in order to evaluate hub genes [10]. In the platinum-based therapy resistant modules, the genes with ∣GS | >0.6 and MM > 0.7 were defined as the hub genes. The genes' relationships of edges and nodes between each other in the hub gene module were exported in a form that could be utilized for Cytoscape visualization. The relationships with weight larger than 0.05 were input into Cytoscape for a PPI network analysis [11]. With the aid of the network analysis function of Cytoscape, the data of degrees of each gene could be gained. Genes with degree larger or equal to 5 were defined as the hub genes in the PPI network. The hub genes which met the screening conditions in both the WGCNA and PPI network analysis would be the final hub genes.

### 2.5. Functional Enrichment Analysis

The genes of the hub gene modules were inputted to the R software for GO enrichment analysis with “enrichGO” function and “http://org.hs.eg.db” database [12]. The genes' IDs for GO analysis were obtained from the “org.Hs.egSYMBOL2EG” database, and the gene without id was cut off. The enrichment pathways were cut off if their *p* value or adjusted *p* value was larger than 0.05 under the condition of “pvalueCutoff = 0.05 and qvalueCutoff = 0.05.” Then, the bar plot of enriched pathways which was set to show the top ten pathways could be obtained. After a gene pathway relative circle diagram to show the genes in top pathways was made, the gene was ordered and colored by |GS|, and the terms would be set to 5 to show the top 5 pathways.

On the other side, in order to determine the functions or effects form the hub gene, the expression level of the hub gene with the highest |GS| would be the standard to make a Gene Set Enrichment Analysis (GSEA) [13] with GSE7880. The median expression level of this gene was calculated; then, the samples with larger expression would be defined as the high-expression group and the others as the low-expression group. Using the GO biological process (BP) database in GSEA software, we would obtain the first 150 pathways and then screen the cancer relative functions.

### 2.6. Verification by Prognosis Analysis

The top four hub genes with a high |GS| were verified by prognostic analysis of hub genes in LUAD on the Kaplan-Meier (KM) website (http://kmplot.com/analysis/). After starting the KM plot for lung cancer and querying the affy id for the hub gene, we chose to illustrate the survival analysis through overall survival (OS). Patient split was set to “auto select best cutoff”. The probe was set to “the user selected probe set.” In addition, the cox regression was set to “univariate” and the array quality control was set to “exclude biased array.” With the other parameters set to “all,” the Kaplan plot was presented.

## 3. Results

### 3.1. Construction of Coexpression Network and Identification of Hub Gene Modules

**Figure 1:**
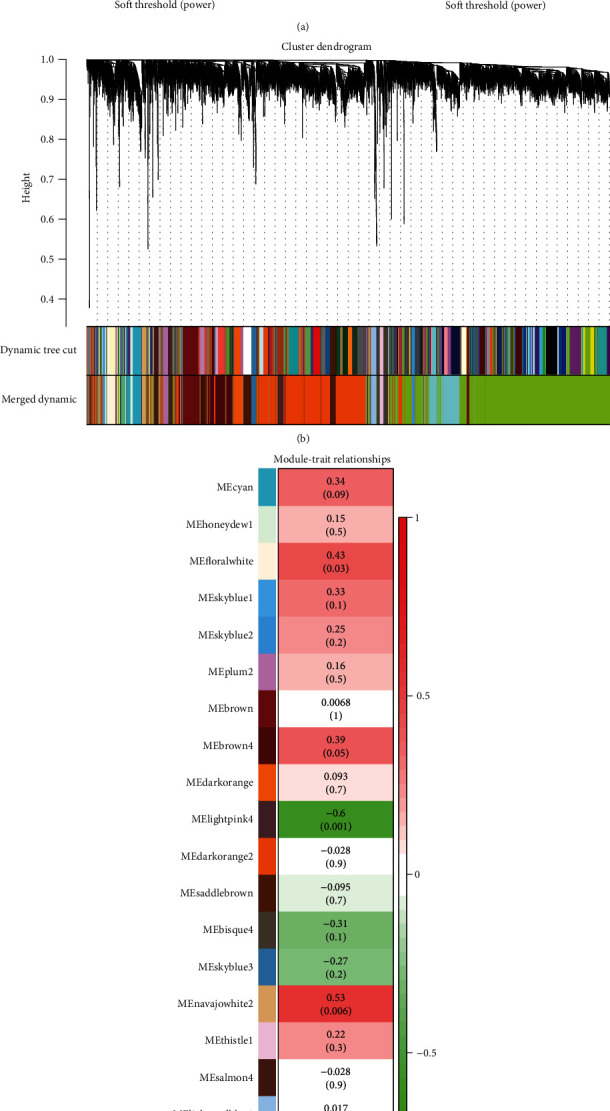
Weighted gene coexpression network (WGCNA): (a) scale free fit index and mean connectivity for different soft threshold power values (these figures are used to identify the optimal power value); (b) the dynamic tree and merged cluster dendrogram; (c) heat map of the correlation level between modules and platinum-based therapy chemoresponse level.

To obtain the gene trait relationship and gene coexpression level, the weighted gene coexpression network was constructed. 25 samples from dataset GSE7880 with LUAD were included in the coexpression network analysis, 15 of which had progressive disease with platinum-based therapy and 10 did not. Meanwhile, the samples with progressive disease were defined as “No responder” and the other samples were defined as “Responder”.  Figure 1(a) shows the WCGNA R package, where the scale free fit index is horizontal and the larger mean connectivity is the optimal power. Following that, the ab-line was set as *R*^2^ = 0.8, and the first power to touch the ab-line was chosen as the soft threshold. The optimal power value was chosen as 11 according to Figure 1(a). The results of the WGCNA are shown in Figure 1(b) with a clustering tree including 22 identified merged modules.  Figure 1(c) represents correlative level between modules and chemoresponse. Among those modules, the lightpink4 shows the highest positive correlation of -0.6 (*p* = 0.001). Modules of floralwhite, navajowhite2, and paleturquoise also had a correlation larger than 0.4 and a *p* value of less than 0.05. The correlation level and *p* value of the floralwhite module were 0.43 and 0.03, those of the navajowhite2 module were 0.53 and 0.006, and those of the paleturquoise module were 0.42 and 0.04, respectively. Therefore, these three modules were chosen as the hub gene modules for a further study. There were 231 genes in the floralwhite module, 156 genes in the lightpink4 module, 62 genes in the navajowhite2 module, and 597 genes in the paleturquoise module, which were used in the PPI network analysis and GO function enrichment analysis.

### 3.2. Hub Gene Identification

**Figure 2:**
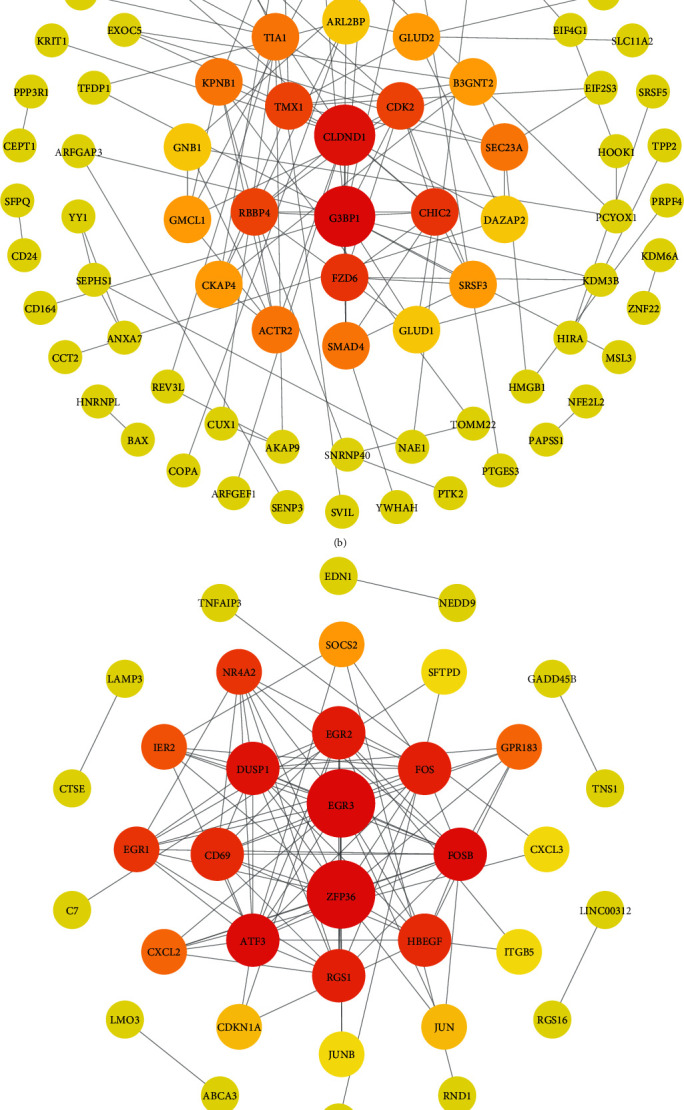
PPI Network for hub gene modules: (a) floralwhite module; (b) lightpink4 module; (c) navajowhite2 module; (d) paleturquoise module. The nodes with large degrees are shown in the center, larger in size and redder in color.

In order to screening significant hub genes, the PPI network was constructed to calculate interaction degrees. In the coexpression network analysis, 41 genes with ∣GS | >0.6 and 588 genes with MM > 0.7 were chosen to participate in the screening, and 23 of these genes met both conditions. With the aid of the network analysis function of Cytoscape, the degrees of genes were obtained. 417 hub genes were selected through the PPI network analysis with a degree of 5 or more.  Figure 2 shows the PPI network of these four modules. Finally, 11 hub genes in four hub gene modules (LY86, ACTR2, CDK2, CKAP4, KPNB1, RBBP4, SMAD4, MYL6, RPS27, TSPAN2, and VAMP2) were chosen as the significant hub genes. The hub genes and their |GS|, MM, and degrees are shown in Table 1.

### 3.3. Functional Enrichment Analysis

**Figure 3:**
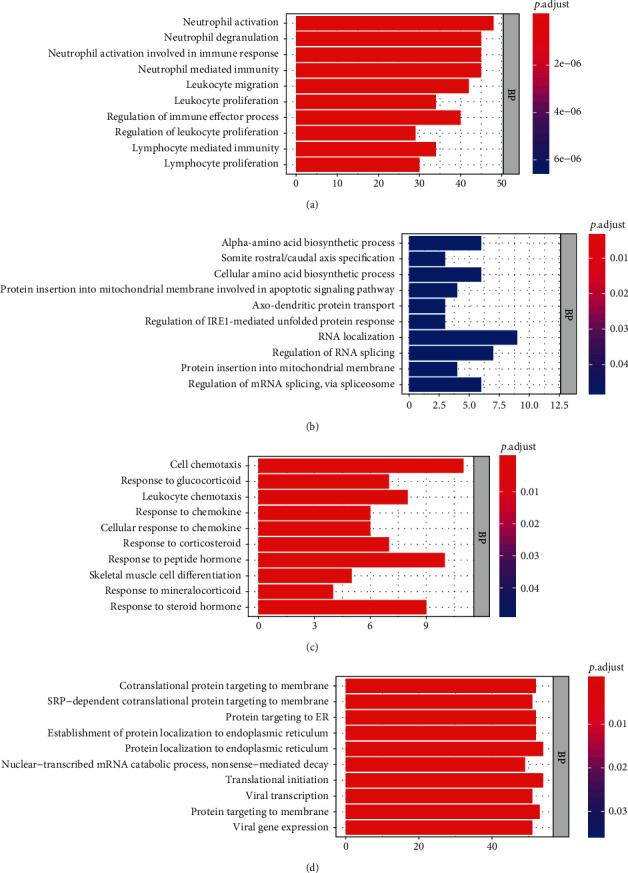
Gene Ontology (GO) Function Enrichment Analysis: (a), (b), (c), and (d) are the enriched biological process (BP) of GO analysis for floralwhite, lightpink4, navajowhite2, and paleturquoise modules, respectively.

**Figure 4:**
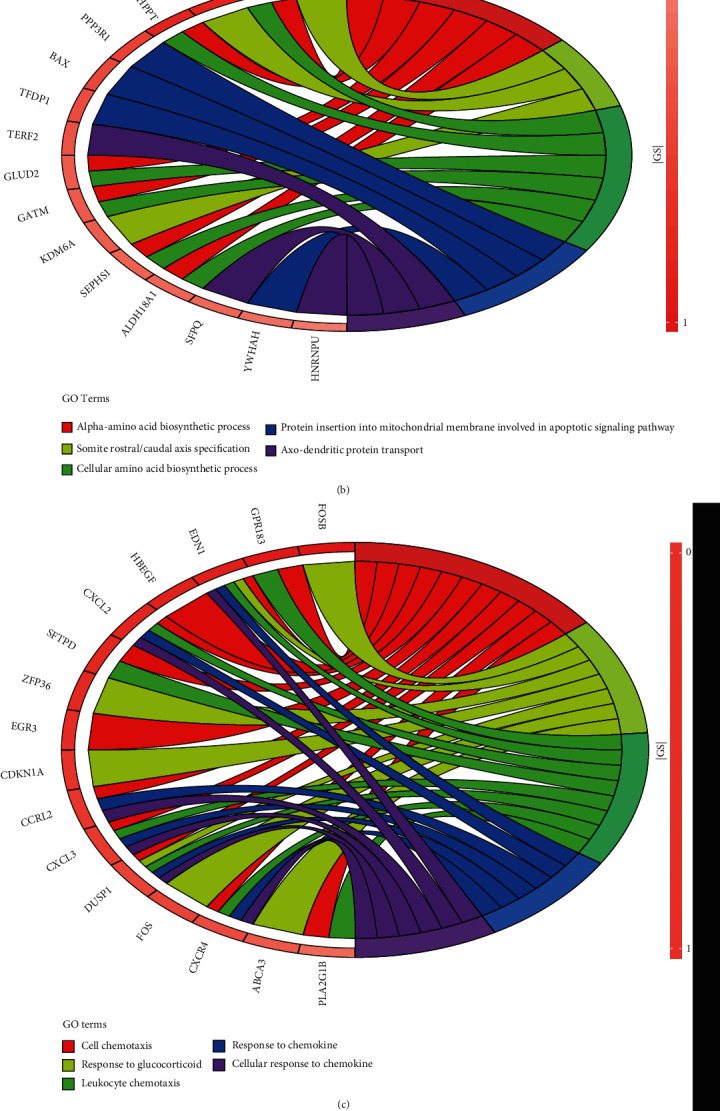
Distribution of genes in the floralwhite module in the first 5 pathways: (a–d) show the circle distribution diagram of floralwhite, lightpink4, navajowhite2, and paleturquoise modules, respectively.

To analysis the function of hub gene modules, the genes in each module were processed by functional enrichment analysis. The GO functional enrichment analysis of the genes in four modules was made with the assistance of R software, including a bar plot (Figure 3) and circle distribution plot (Figure 4). The GO functional analysis was divided into three parts: BP, cell component (CC), and molecular function (MF) [14]. Among these, we focused mainly on the BP part. Figures 3(a) and 4(a) show the results of the GO analysis for floralwhite module genes. It illustrates that the top pathways involved activities of neutrophil functions and the immune system. Neutrophils are the human's first defense against infection, which can cause responses and fight against cancer. Furthermore, the cancer could be detected because of abnormal proliferation, which meant the gene in this module had great influence on the tumors' occurrence [15]. In the lightpink4 module, the top pathways in the BP mostly involved amino acid biosynthesis. Cell proliferation is inseparable from protein synthesis, and the synthesis of alpha amino acids, as the main components of protein, will affect the proliferation and recurrence of tumor cells. In the navajowhite2 module, the top pathways are about chemotaxis and chemokine, which could influence tumor metastasis. In the paleturquoise module, the top pathways are about protein targeting the membrane, which could be a target in drug therapy. In sum, these four modules all have some relationship with cancer recurrence.

**Figure 5:**
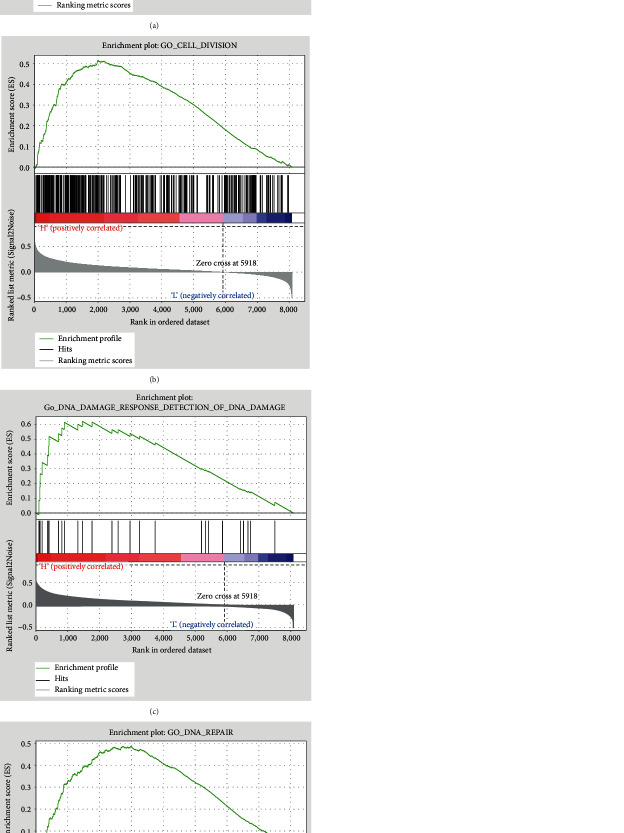
GSEA of RBBP4: (a) positive regulation of DNA replication with *p* = 0.008 and a normalized enrichment score (NES) = 1.57; (b) cell division with *p* = 0.014 and NES = 1.57; (c) response to DNA damage with *p* = 0.027 and NES = 1.161; (d) DNA repair with *p* = 0.024 and NES = 1.57.

Because the gene Retinoblastoma-Binding Protein 4 (RBBP4) had the highest |GS|, we used it as the standard for a gene set enrichment analysis. Two representative diagrams of the enrichment plot are shown in Figure 5. According to Figures 5(a) and 5(b), the genes expressed in the high RBBP4 expression group were enriched in the pathway of positive regulation of DNA replication and cell cycle. That means RBBP4 might have influence on the increase of the DNA replication ability and enhance the cell cycle to act as a factor of tumor recurrence. In Figures 5(c) and 5(d), the high expression group was more sensitive to DNA damage and had a high expression level for the DNA repairing pathway. The mechanism of action of platinum chemotherapy is inducing DNA damage to the cancer cell. Therefore, a high DNA repair ability could cause efficacy lose. Thus, RBBP4 could possess the ability to enhance DNA repairing ability resulting in chemoresistance.

### 3.4. Verification by Prognosis Analysis

**Figure 6:**
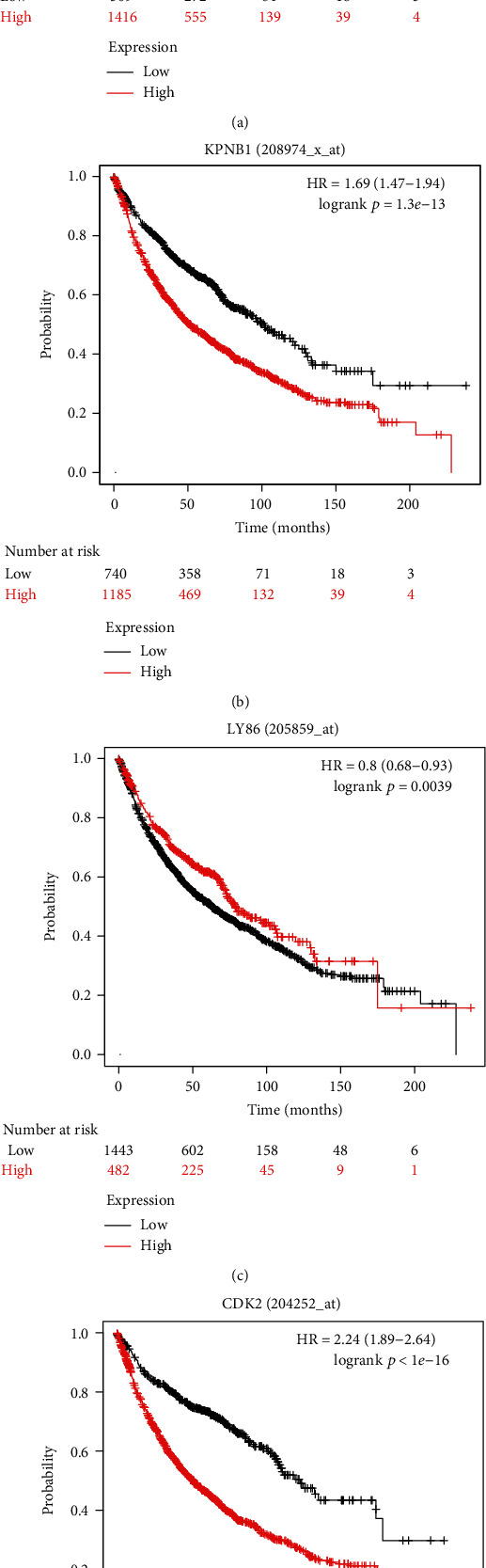
Prognostic analysis of the top four hub genes with the highest GS absolute value: (a) Kaplan plot of RBBP4 (217015_at; GS = −0.73); (b) Kaplan plot of KPNB1 (208974_x_at; GS = −0.72); (c) Kaplan plot of LY86 (205859_at; GS = 0.67); (d) Kaplan plot of CDK2 (204252_at; GS = −0.65).

To demonstrate the significance relationship between hub gene expression and survival, prognosis analysis was processed. The top four genes with the highest |GS| were uploaded to the KM website (http://kmplot.com/analysis/) to retrieve survival information. We started a KM plot for lung cancer and queried the affy id for the four genes. With the settings prepared as described in Materials and Methods, the Kaplan plot was obtained and the results are shown in Figure 6. The number of risks of each group is shown under the plot. The red group is the high-expression group, and the black group is the low-expression group. According to the Kaplan plot the higher expression of RBBP4, KBNP1 and CDK2 could cause a lower survival rate. The correlative value of |GS| for these genes was also negative to the chemoresponse. Therefore, RBBP4, KBNP1 and CDK2 could be a function enhancer or biomarker for recurrence of LUAD with platinum-based therapy. While the higher expression of LY86 caused a higher survival rate, the |GS| for LY86 was positive. Thus, LY86 could have a positive effect on chemoresponse.

## 4. Discussion

LUAD accounts for a large proportion of lung cancers [3]. Early diagnosis of LUAD is difficult, and most patients are already in the local advanced stage or have distant metastasis at diagnosis, which is prone to recurrence and poses a great challenge to treatment and prognosis. Platinum-based chemotherapy has been a great contribution to treatment [16] and plays a very important role [17]. Even after early diagnosis, in addition to stage I patients, patients usually receive adjuvant chemotherapy in order to prevent postoperative recurrence. However, due to metastasis of tumor cells and the resistance to chemotherapy drugs, the recurrence rate of tumor remains high. Therefore, it is necessary to identify possible biomarkers in order to find effective targets to inhibit tumor recurrence and reduce the recurrence rate. In this study, we used the GEO dataset GSE7880. This dataset was uploaded by Rohrbeck et al. in 2007 and contains gene expression data from patients with NSCLC. We went back to this data set and used a new analysis to make it more valuable. 25 gene expression samples of patients with LUAD were screened, and they were divided into two groups according to the chemotherapy response for a WGCNA. Through the fitting of traits, we obtained four gene modules that were highly correlated with the chemotherapy response. In combination with a PPI network analysis, we screened 11 key genes that were highly correlated with the response to platinum-based chemotherapy in LUAD. The functional directions of four gene modules were determined by a GO functional enrichment analysis, namely, “immune system,” “cell amino acid and chromatin activity,” “cell chemotaxis,” and “targeted membrane protein.” They were associated with “cancer cell clearance,” “cell proliferation,” “cell metastasis,” and “drug targets,” respectively, which made these genes valuable for future research regarding the treatment of LUAD. As the gene RBBP4 most related to chemical reaction traits in the fitting, we used it for GSEA enrichment analysis and found that DNA replication and cell division were hyperactive in its high expression group. Therefore, we speculated that RBBP4 could be used as a biomarker or target of platinum resistance or tumor recurrence for subsequent studies. In addition, in the analysis of prognosis, the high expression of RBBP4 resulted in a reduced survival rate, which also demonstrated its negative effect in cancer recovery.

Cisplatin is a kind of metal platinum complex commonly used at present. The platinum atom in the molecule is of great significance for its antitumor effect. It can cross-link with DNA strands, showing cytotoxic effects. The solution passes through the charged cell membrane in the body without a carrier transport. Due to the low concentration of chloride ions in the cell, the chloride ions are replaced by water and have a positive charge, which acts like an alkylating agent double functional group, and can combine with the DNA bases in the nucleus to form three forms of cross-linking, causing DNA damage, destroying DNA replication and transcription, and inhibiting the synthesis of RNA and protein at high concentration [18].

One significant mechanism for cisplatin chemoresistance is the on-target resistance. The cisplatin resistant cells acquire the ability to repair adducts or become able to tolerate unrepaired DNA [19]. The data of lightpink4 which contains the hub gene RBBP4 with the GO function of DNA repair also shows an opposite of chemoresponse. According to GSEA analysis, RBBP4 can increase the DNA damage detecting and repairing ability, which can contribute to on-target resistance of the cancer cells. Meanwhile, currently, RBBP4 which is also abbreviated as RbAp48 was regarded as a regulative factor for DNA repair. RbAp48 was firstly reported as a negative regulator for Ras in yeast [20]. Then, in 1996, RbAp48 was discovered to be one of the three subunits of the chromatin assembly factor 1 [21, 22]. It is a component of the histone deacetylase complexes and is involved in chromatin remodeling [23, 24] which could contribute to DNA repairing and cell division. It was also reported that it could decrease temozolomide, an anticancer drug with the action of inducing DNA methylation damage, and sensitivity in regulating DNA repairing proteins [25]. In addition, RBBP4 was also reported as a significant differently expressed gene between oropharyngeal squamous cell carcinoma patients and patients without cancer [26] in 2009. Combined with the DNA damage repair function of RBBP4 and its high expression in cancer, we can infer that it must also play an important role in the resistance of platinum chemotherapy. However, there have been insufficient reports on the link between this gene and platinum-based chemotherapy resistance in LUAD. Starting from a real clinical sample data set, this research found abnormal expression of this gene in the LUAD resistance group by comparing the drug resistance group and the response group, which provided statistical evidence for its important role in the resistance of platinum chemotherapy in LUAD.

In this study, a variety of bioinformatics analysis methods were used, and the gene and protein levels were rigorously analyzed and screened. However, it is still lacking in cell biology experiments, molecular biology experiments and other physical experiments. There are some limitations in evidence based entirely on data. Therefore, in future research, we will further demonstrate the drug resistance effect of RBBP4 by combining it with solid experiments, explore its drug resistance mechanism, and explore the possibility of it becoming a biomarker and target.

## Figures and Tables

**Table 1:** Hub genes in modules.

Gene name	Module color	Gene trait significance (GS > 0.6)	Gene module membership (MM > 0.7)	Degree (degree > 5)
LY86	floralwhite	0.6679241	0.8069434	17
ACTR2	lightpink4	-0.6020996	0.9098197	6
SMAD4	lightpink4	-0.6104894	0.7267321	6
CKAP4	lightpink4	-0.6220552	0.7872074	5
CDK2	lightpink4	-0.6548132	0.8920717	8
KPNB1	lightpink4	-0.7198137	0.8535104	6
RBBP4	lightpink4	-0.7327022	0.8804876	8
RPS27	paleturquoise	0.6380246	0.7398567	32
TSPAN2	paleturquoise	0.6255706	0.7910007	25
MYL6	paleturquoise	0.6124084	0.7494138	37
VAMP2	paleturquoise	0.6065676	0.778348	27

## Data Availability

The underlying data supporting this research was the micro array matrix downloaded from GSE7880, which was uploaded by Rohrbeck et.al in 2007 and can be obtained from GEO database (https://www.ncbi.nlm.nih.gov/geo/query/acc.cgi?acc=GSE7880).
